# Trilateral Intercomparison of Photometric Units Maintained at NIST (USA), NPL (UK), and PTB (Germany)

**DOI:** 10.6028/jres.104.003

**Published:** 1999-02-01

**Authors:** Yoshi Ohno, Teresa Goodman, Georg Sauter

**Affiliations:** National Institute of Standards and Technology, Gaithersburg, MD 20899-8442 USA; National Physical Laboratory, Queens Road, Teddington, Middlesex TW11 0LW UK; Physikalisch-Technische Bundesanstalt, Abteilung Optik, Photometrie, Postfach 33 45, 38023 Braunschweig, Germany

**Keywords:** illuminance, intercomparison, luminous flux, luminous intensity, luminous responsivity, photometer, photometry, units

## Abstract

A trilateral intercomparison of photometric units between NIST (USA), NPL (UK), and PTB (Germany) has been conducted to update the knowledge of the relationship between the photometric units disseminated in the three countries. The luminous intensity unit (cd), the luminous responsivity scale (A/lx), and the luminous flux unit (lm) maintained at each laboratory were compared by circulating transfer standard lamps and photometers. The results showed that the relative luminous intensity values, with respect to the average, measured by NIST, NPL, and PTB were 1.0014, 1.0021, and 0.9966; the relative inverse values of the luminous responsivity (corresponding to illuminance) were 1.0023, 1.0011, and 0.9965; the relative luminous flux values were 0.9994, 1.0034, and 0.9972, respectively. The results agreed within the stated uncertainties of the units maintained at the three laboratories.

## 1. Introduction

A trilateral intercomparison of photometric units between the National Institute of Standards and Technology (NIST, USA), the National Physical Laboratory (NPL, UK), and the Physikalisch-Technische Bundesanstalt (PTB, Germany) was conducted to update the knowledge of the relationship of the photometric units disseminated in the three countries. This was the first photometric intercomparison between NIST and NPL since the Comité Consultatif de Photométrie et Radiométrie (CCPR) intercomparison in 1985 [1] and between PTB and NIST since 1993 [2]. This intercomparison was stimulated by the new realization of the lumen by NIST in 1995 using the absolute integrating sphere method [3].

The luminous intensity unit (cd), the luminous responsivity scale (responsivity of a photometer for illuminance; unit: A/lx), and the luminous flux unit (lm) maintained at the three laboratories were compared by circulating transfer standard lamps and standard photometers among the three laboratories during the period from June 1995 to November 1997. Seven luminous intensity standard lamps, eight luminous flux standard lamps, and three standard photometers, prepared by the three laboratories, were used as transfer standards. All the transfer standards were hand-carried between the laboratories during the intercomparison.

All the laboratories realize their luminous intensity units based on cryogenic radiometers [4–6]. The methods of realization of the units used by the three laboratories are similar but with small differences, which are discussed in the next section. NIST maintains the unit via a group of standard photometers. NPL maintains the unit via both standard lamps and standard photometers. PTB maintains the unit via a group of standard lamps.

This trilateral intercomparison was performed a few years before the 1998 CCPR intercomparison of photometric units. The results not only provided the updated relationship of photometric units between the three laboratories, but also offered some preliminary information on the stability of transfer standards to be used for the next CCPR intercomparison.

## 2. Realization and Maintenance of the Photometric Units at Each Laboratory

### 2.1 NIST

The NIST luminous intensity unit has been realized annually based on the absolute cryogenic radiometer since 1992. The unit is realized and maintained via a group of eight standard photometers calibrated for luminous responsivity (A/lx). The NIST standard photometers are composed of a silicon photodiode, *V*(λ)-correction filter, and an aperture, and are also equipped with a temperature sensor that allows for a correction for the change of photometer temperature. The luminous intensity *I*_v_(cd)^1^ of a light source is derived from the photocurrent *y*(A) and the source-to-photometer distance *d* (m) according to the equation

$$I_{v}=K_{m}\cdot \frac{d^{2}}{A}\cdot \frac{\int_{\lambda }S(\lambda )V(\lambda )d\lambda }{\int_{\lambda }S(\lambda )s(\lambda )d\lambda }\cdot y\cdot c_{t}\cdot c_{s},$$ (1)

where *K*_m_ = 683 lm/W, *S*(λ) is the relative spectral power distribution of a light source being measured, *s*(λ) is the absolute spectral responsivity (A/W) of the photometer, *V*(λ) is the spectral luminous efficiency function, and λ is the wavelength (m). *A* is the area (m^2^) of the aperture, *c*_s_ is the correction factor for the spatial nonuniformity of responsivity over the aperture, and *c*_t_ is the correction factor for the photometer temperature. The values of *s*(λ) and *c*_s_ are determined annually by the NIST Spectral Comparator Facility (SCF) [7] that is traceable to the NIST High Accuracy Cryogenic Radiometer (HACR) [8]. The relative expanded uncertainty (*k* = 2) of the NIST luminous intensity unit is 0.39 %. Further details of the realization process are described elsewhere [4]. The luminous intensity and the luminous responsivity measurements in this trilateral intercomparison were based on the NIST candela realized in 1995 and 1996.

The NIST luminous flux unit has been derived from the NIST luminous intensity unit using the Absolute Integrating Sphere Method since 1995 [3]. A 2.5 m integrating sphere is now used to realize the unit and to conduct substitution measurements. The unit is realized via a group of 16 luminous flux standard lamps. Eight of these lamps are used as the primary reference standards and the rest for routine calibration measurements. The relative expanded uncertainty (*k* = 2) of the NIST luminous flux unit is 0.53 %. The luminous flux measurements in this trilateral intercomparison were based on the NIST lumen realized in 1995.

### 2.2 NPL

A full realization of the NPL candela was performed in 1985 based on two specially designed photometers, calibrated by reference to the NPL cryogenic radiometer [5]. Since 1985 the unit has been maintained by four batches of incandescent reference lamps (16 lamps in total) and by the two reference photometers. The lamps and photometers have been intercompared approximately annually and the calibration of the reference photometers has been periodically checked by reference to the NPL cryogenic radiometer. These measurements have all confirmed that the luminous intensity unit maintained by NPL has been unchanged since the CCPR intercomparison in 1985, within the relative uncertainty of the realization (0.19 %, *k* = 2).

The reference photometers used were developed at NPL. They consist of a silicon photodiode, a four-element glass filter for *V*(λ) correction, and an aperture and are housed in temperature-controlled water jackets. The luminous intensity of a light source *I*_v_ is derived from the measured photocurrent *y* of the photometer according to the equation

$$I_{v}=k_{m}\frac{d^{2}}{A}\cdot \frac{F}{s(555)}\cdot y,$$ (2)

where *K*_m_ = 683 lm/W, *A* is the area of the aperture, *d* is the source-to-photometer distance, and *s*(555) is the absolute responsivity (A/W) of the photometer at a wavelength of 555 nm. *F* is a correction factor to allow for imperfections in the match between the photometer responsivity function *s*(λ) and the *V*(λ) function for the source being measured, and is given by

$$F=\frac{\int_{\lambda }S(\lambda )V(\lambda )d\lambda }{\int_{\lambda }S(\lambda )s_{\text{rel}}(\lambda )d\lambda },$$ (3)

where *S*(λ) is the relative spectral power distribution of a light source being measured, and *s*_rel_(λ) is the relative spectral responsivity of the photometer, normalized to 1.000 at 555 nm. The 1985 realization involved calibrating *s*_rel_(λ) and *s*(555) by direct reference to the NPL spectral responsivity scale as maintained by large area silicon photodiodes that were calibrated against the NPL cryogenic radiometer. The unit was established via four groups of NPL/GEC^2^ lamps (now known as NPL/Polaron LIS lamps) and Osram type Wi41/G lamps, operated at distribution temperatures ranging from 2800 K to 2856 K. The overall relative expanded uncertainty of the realization of the luminous intensity unit with both types of lamp was assessed as 0.19 % (*k* = 2). As described earlier, these groups of lamps and the reference photometers have been used to maintain the luminous intensity unit since 1985. By conducting regular comparisons between them and by periodic recalibration of the photometers by reference to the cryogenic radiometer, it has been confirmed that the NPL luminous intensity unit has remained constant during this time. Further details of the realization of the NPL candela are published in [5].

The NPL luminous flux unit was realized in 1985 using an NPL-designed goniophotometer with a diameter of approximately 3.5 m. The goniophotometer was of a two-axis type, with a photometer head rotating in one longitudinal plane around the lamp mounted on the center of rotation. After each longitudinal scan, the lamp was rotated by a small angular increment about its vertical axis and the process repeated until the full 360° had been sampled. The photometer head was calibrated against luminous intensity standard lamps mounted in the center of rotation, these lamps being calibrated against the cryogenic radiometer-based scale described previously. Laser beams intersecting at the center of rotation were used to align the standard lamps. Where necessary, depending on the type of luminous flux lamp calibrated, corrections were applied for a small shadow cast by the lamp rotation mechanism. The NPL luminous flux unit was established via two groups of luminous flux reference standard lamps (NPL/Polaron LF200 and LF500) with a relative expanded uncertainty (*k* = 2) of 0.35 %. Since 1985 regular checks on the long term maintenance of the luminous flux unit at NPL using these lamps have been made, confirming that the NPL lumen, like the NPL candela, has remained unchanged since the CCPR intercomparison in 1985. Further details of the NPL realization of the lumen are given in Refs.[5 and 9].

Routine flux measurements at NPL are currently performed using the NPL 4.6 m integrating sphere and measurements in this trilateral intercomparison were carried out using this sphere, by reference to the luminous flux reference standard lamps established on the goniophotometer. Since this intercomparison, the goniophotometer described above has been replaced by a new version, which will ultimately be used for all luminous flux measurements at NPL.

### 2.3 PTB

The luminous intensity unit at PTB was first realized in 1980 based on radiometric power measurements with absolute radiometers, and the unit has been maintained by a batch of 23 incandescent lamps (Toshiba 5 A, 14 cd, 2042 K). Since then, the luminous intensity unit has been realized annually with two reference photometers based on the absolute radiometers (recently an absolute cryogenic radiometer) [6]. The luminous intensity of a light source *I*_v_ is determined from the measured photocurrent *y* of the photometer according to the equation:

$$I_{v}=K_{m}\frac{d^{2}}{\Omega_{0}}\cdot \frac{F}{s_{a}(555)}\cdot y$$ (4)

where *s*_a_(555) is the absolute irradiance responsivity [AW^−1^ m^2^] at a wavelength of 555 nm, and *Ω*_0_ is the unit solid angle [sr]. The color-correction factor *F*, defined in Eq. (3), is obtained as a function of the distribution temperature of the light source being measured. Since the photometers used in the realization are temperature-controlled, no correction is needed for variations of the photometer temperature.

The unit realized annually via the reference photometers, however, has shown variations about the unit maintained via the reference lamps (within the uncertainty of realization), while the reference lamp unit has remained more stable. Therefore, the reference lamp unit has been maintained with no adjustment. This unit is transferred to batches of working standard lamps (Osram Wi41/G). The relative expanded uncertainty (*k* = 2) of the maintained unit of luminous intensity at PTB is 0.4 %. Further details of the realization process for the PTB candela are described in Ref. [6].

The PTB luminous flux unit is derived from the luminous intensity unit by goniophotometric measurements and maintained by several batches of incandescent lamps. The PTB goniophotometer has a diameter of 5 m and three axes of rotation that enables measurement without moving or turning the lamp under test at any burning position. The total luminous flux is obtained by spatial integration of illuminance. The photometer head of the goniophotometer is periodically calibrated for luminous responsivity against the PTB luminous intensity working standard lamps. The relative expanded uncertainty (*k* = 2) of the maintained unit of luminous flux at PTB is 0.6 %. Further details of the realization process for the PTB lumen are described in Ref. [10]. The PTB photometric units have remained unchanged since the CCPR intercomparison in 1985.

## 3. Intercomparison Scheme

The measurements for this trilateral intercomparison were performed between June 1995 and November 1997. The measurement scheme of the intercomparison is shown in Table 1. The transfer artifacts were seven luminous intensity standard lamps (three prepared by NIST, two by NPL, two by PTB), three standard photometers (one prepared by each laboratory), and eight luminous flux standard lamps (four prepared by NIST, two by NPL, two by PTB). The details of these artifacts are described in the following sections.

All the artifacts were hand-carried between laboratories. First, the transfer lamps and photometers from NIST and PTB were measured at these laboratories before transportation to NPL in June 1995. All the transfer artifacts were measured at NPL in November 1995. Immediately after that, the artifacts were brought to PTB and measured there with N. Pearce from NPL participating in the measurements, and then, brought back to NPL. After re-measurement at NPL in June 1996, all the artifacts were carried to NIST and were measured in July 1996, when G. Sauter from PTB and N. Pearce from NPL joined the measurements at NIST. After their visit to NIST, the NIST lamps (except the two large flux lamps) were carried to PTB again and measured in September 1996 when Y. Ohno from NIST visited PTB and carried the lamps back to NIST. The NIST intensity lamps were measured in October 1996 and flux lamps in August 1997 (delayed due to construction of a new sphere). The artifacts from NPL and PTB were carried back to NPL and measured at NPL by March 1997. Finally, the artifacts from PTB were carried back to PTB from NPL and measured by November 1997 to complete the intercomparison. Therefore, all the transfer lamps and photometers were circulated to all three laboratories, and were measured by the laboratory that prepared them before and after circulation to the other two participants.

## 4. Luminous Intensity Comparison

### 4.1 Transfer Lamps

The three luminous intensity transfer lamps prepared by NIST were Osram Sylvania 1000 W FEL type quartz halogen lamps (85 V, 7 A, 2856 K), potted on a bi-post base. The two lamps prepared by NPL were NPL/Polaron LIS (12 V, 25 A, 2856 K), gas-filled incandescent lamps. The two transfer lamps prepared by PTB were Osram Wi41/G (30 V, 6 A, 2800 K), gas-filled incandescent lamps. All the lamps were operated at a constant dc current. The NIST and PTB lamps were operated with a fixed polarity; the NPL lamps were operated with the polarity alternated at every burning according to normal NPL practice (this reduces the aging rate with this type of lamp). The voltage across the lamps was measured to check for unexpected changes which might indicate that damage had occurred during transit, for example. These types of lamps have been used at each laboratory over many years and their reproducibilities were known to be better than 0.1 %. The aging rates (relative changes of luminous flux due to burning of the lamp) of the FEL type, the NPL/Polaron LIS type, and the Wi41/G type lamps, are approximately −0.01 %/h, −0.01 %/h, and −0.03 %/h, respectively. The distribution temperatures of the lamps were measured but used only for spectral mismatch correction purposes.

### 4.2 Measurements at Each Laboratory

At NIST, the luminous intensity measurements were based on the NIST candela realized in 1995 and 1996. Three of the NIST standard photometers were used to measure the luminous intensity transfer lamps. Measurements were performed using the NIST photometry bench [4] with a photometric distance of approximately 3.5 m. The lamp current was measured with a shunt resistor (0.1 Ω) for all the lamps. The shunt resistor was calibrated at 0.5 A, 5 A, 10 A, and 25 A by the NIST Electricity Division, and the resistance was fitted using a polynomial function. The lamp current was set with a relative expanded uncertainty of 0.01 % (*k* = 2), automatically controlled with a feedback system, and had a relative stability of ±0.002 %. Further details of the NIST measurement facility are found in [11].

At NPL, measurements were made against the NPL reference lamps described in Sect. 2.2 above, at a distance of approximately 2.5 m. The lamp current was monitored with a shunt resistor (0.01 Ω for the NPL/Polaron lamps or 0.1 Ω for the Osram lamps) and a high-accuracy digital voltmeter. The resistors were immersed in an oil bath to ensure stable operating conditions. The calibrations of the resistors and the digital voltmeter were traceable to NPL electrical standards. The relative expanded uncertainty in the current supplied to the lamps was better than 0.01 % (*k* = 2) in all cases and was stabilized to better than ±0.001 %.

At PTB, measurements were made against the PTB luminous intensity working standard lamps at a distance of approximately 6 m. The power supply allowed the current to the lamp to be set with a resolution of better than 0.002 % of the measured value and was stabilized to better than ±0.001 % of the current during operation of the lamp. The relative expanded uncertainty of the electrical quantities was estimated to be 0.02 %.

At each laboratory, the lamps were aligned using the same method and nearly the same procedures. The FEL lamps were aligned using an alignment jig (a flat glass plate mounted on a bi-post base) and a laser beam for autocollimation. This alignment jig was transported together with the FEL lamps. Four-pole FEL lamp sockets of the same design were used by the three laboratories. The NPL/Polaron LIS lamps were aligned by autocollimation of a laser beam reflected from the flat window of the lamp, and their position adjusted so that the filament was centered about the optical axis. A special socket for the LIS lamp was supplied by NPL for use during the intercomparison. The Wi41/G lamps were aligned using two telescopes, one along the optical axis, the other perpendicular to the optical axis. The tilt and rotation of the lamp were adjusted so that the filament was parallel with the fiducial line of the side telescope and the lamp position was adjusted so the filament was centered about the optical axis. Distance was measured from the mean plane of the filament for the Wi41/G and the NPL/Polaron LIS lamps; for the FEL lamps it was set using the alignment jig. The three laboratories conducted measurements at their normal working distances (3.5 m at NIST, 2.5 m at NPL, 6 m at PTB), with the exception that the NIST lamps were set at 3.5 m by all three laboratories. Measurements made previously at NPL and PTB have shown that for the Wi41/G and NPL/Polaron LIS lamps consistent results are obtained at all distances within the range 2.5 m to 6 m (i.e., these lamps obey the inverse square law at these distances).

### 4.3 Results

Table 2 shows the results of the luminous intensity measurements by the three laboratories. Each luminous intensity value is the result of two or more measurements in separate burnings of each lamp. The ratios of the luminous intensity values relative to the average of the three laboratories are analyzed in Table 3. The uncertainty of the intercomparison was evaluated statistically based on the reproducibility of measurements of transfer artifacts. The column “*σ* of the mean” shows the relative standard deviation of the mean of the measured values for each lamp at each laboratory, to which the typical reproducibility of measurements of these transfer lamps (0.05 %) has been added in quadrature. A value for *σ* was assessed only when the lamp was measured more than twice during the intercomparison at the same laboratory. The values of *σ* for all three laboratories are averaged and denoted as *σ*_1_ in the last column of the table. In the bottom rows, the ratios for the seven lamps for each laboratory are averaged, and the values of *σ*_1_ for all the lamps are averaged and denoted as *σ*_2_. The relative expanded uncertainty of the comparison result, *U* (*k* = 2), is twice *σ*_2_. The changes in the lamps during the comparison are assessed by the differences between the first and the last values measured by the laboratory that prepared the lamps. The relative differences (the last value minus the first value), based on the averages of the lamp groups, were found to be 0.05 % (NIST lamps), +0.04 % (NPL lamps), and +0.05 % (PTB lamps), the maximum for any individual lamp being +0.33 % of the measured value.

Table 4 shows the analysis of the measured lamp voltages. The uncertainty was analyzed in the same manner as in Table 3 except that the uncertainty for the typical reproducibility of measurements is not included as it is normally negligible. The results show no notable differences in the electrical measurements by the three laboratories, and also indicate no notable changes in the electrical characteristics of the lamps during the comparison campaign. The significant difference between the voltage measurements made at PTB and those made at NPL and NIST for the NPL/Polaron LIS lamps was due to a difference in the placement of the voltage probes and was not indicative of a change in the operating characteristics of these lamps, as confirmed by the good reproducibility of the luminous intensity measurements throughout the comparison.

From the results obtained, the relationship between the magnitude of the luminous intensity units maintained by the three laboratories is; NIST candela / NPL candela = 1.0007, NPL candela / PTB candela = 0.9945, and PTB candela / NIST candela = 1.0048. With the stated relative expanded uncertainties (*k* = 2) of the luminous intensity units of the three laboratories ranging from 0.2 % (NPL) to 0.4 % (PTB and NIST), the uncertainty bars of the three laboratories overlap with one another. The relative range of variations of the magnitude of the units among the three laboratories (0.55 %) is a considerable improvement over the results of the 1985 CCPR intercomparison (0.9 %) [1].

## 5. Luminous Responsivity Comparison

### 5.1 Transfer Photometers

Three transfer standard photometers, one each from each laboratory, were used in the luminous responsivity comparison. The NIST transfer photometer was a non-diffuser type, the same type as the NIST standard photometers used in the realization of the candela [4], and was equipped with a temperature sensor and built-in current-to-voltage converter. Its luminous responsivity was corrected for differences in photometer temperature and the results given apply for a temperature of 25 °C. The NPL transfer photometer was a commercially-manufactured non-diffuser type with a 12 mm aperture, which was not equipped with a temperature monitor and was not temperature-controlled. The PTB transfer photometer was an LMT model P10F0T, which is a temperature-controlled type (maintained at 35 °C) equipped with an opal diffuser and a 10 mm aperture. An external, calibrated current-to-voltage converter was used with the NPL photometer and the PTB photometer.

### 5.2 Measurements at Each Laboratory

At all the laboratories, the luminous responsivity was determined for the CIE Illuminant A, using standard lamps operated at a distribution temperature of 2856 K. The measurements at NIST were based on the NIST candela realized in 1995 and 1996. The luminous responsivity was measured against three of the NIST standard photometers at distances of 2.5 m and 3.5 m, at illuminance levels of approximately 170 lx and 90 lx, respectively. An FEL-type working standard lamp operated at 2856 K was used. At NPL, the luminous responsivity of the photometers was measured against the NPL luminous intensity reference lamps (2856 K) at a distance of approximately 2.5 m. At PTB, the measurements were made against the PTB luminous intensity working standard lamps operated at 2856 K, and at two distances around 3.5 m.

### 5.3 Results

Table 5 shows the results of the luminous responsivity measurements by the three laboratories. Each value is a result of two or more measurements with realignment of the photometer heads. Table 6 shows the ratios of the luminous responsivity values relative to the average of the three laboratories, together with the statistical uncertainty budget. The data are analyzed in the same manner as given in Table 3. The relative expanded uncertainty of the results *U* (*k* = 2) is determined to be 0.23 %, much higher than the luminous intensity results. A relatively large change of the photometer supplied by NPL contributed to the increased uncertainty of the comparison and was probably due to the lack of effective temperature monitoring and control. This photometer was a commercial device of a type that is no longer manufactured. If the NPL photometer is excluded, the relative expanded uncertainty reduces to 0.15 %, which is comparable with that for the luminous intensity results. The relative changes of the photometers during the comparison (the last value minus the first value measured at the laboratory that prepared the photometer) were −0.19 % (NIST photometer), −0.63 % (NPL photometer), and +0.12 % (PTB photometer). The PTB photometer was set up incorrectly at NPL in November 95 and no data are therefore given for this entry in the table.

In order to compare the results with the luminous intensity comparison results, the inverse values of the relative luminous responsivity values (corresponding to illuminance) of NIST, NPL, and PTB were calculated to be 1.0023, 1.0011, and 0.9965, respectively, with the relative range of variation being 0.56 %. These results agree with the results of the luminous intensity comparison to within 0.11 % of the latter. The variation among the three laboratories is within the uncertainties of the luminous intensity units maintained at the three laboratories.

## 6. Luminous Flux Comparison

### 6.1 Transfer Lamps

Two Polaron LF200 lamps (90 V, 2 A, 2730 K) and two Osram opal-bulb 40W lamps (24 V, 1.7 A, 2730 K) were prepared by NIST, two Polaron LF300 lamps (120 V, 2.4 A, 2750 K) were prepared by NPL, and two Osram Wi4 (24 V / 100 W—frosted bulb, 2850 K) were prepared by PTB. Typical aging rates for these luminous flux lamps are about −0.02 %/h. All the lamps had E27 screw bases, and were operated in the base-up position at specified currents with the voltages monitored. All the laboratories used a four-pole socket to mount the lamps and to measure the lamp voltages. The distribution temperature values were provided by the laboratories that prepared the lamps for spectral mismatch correction purposes only, and were not measured during the luminous flux intercomparison.

### 6.2 Measurements at Each Laboratory

At NIST, the luminous flux transfer lamps were measured against four of the NIST luminous flux working standard lamps with a substitution method using the NIST 2 m integrating sphere as described in Ref. [11]. Each lamp was measured twice in separate burnings. Each measurement took 1 min after stabilizing the lamp. The self-absorption correction factors of all the lamps were measured and applied. Spectral mismatch corrections were applied, based on the distribution temperatures of the lamps. The electrical instrumentation is similar to that used in the luminous intensity measurement. The current was set to a specified value with a relative expanded uncertainty of 0.01 % (*k* = 2) and stabilized to within 0.002 %.

At NPL, the measurements were carried out using the 4.6 m integrating sphere that was calibrated on each occasion using the luminous flux reference standard lamps established by the goniophotometer described in Sec. 2.2. Each transfer lamp was measured twice in separate burnings. Each measurement took one minute after stabilizing the lamp. The self-absorption correction factors of all the lamps were measured and applied. The electrical instrumentation for lamp operation is similar to the one described for the luminous intensity measurements at NPL.

At PTB, the measurements were conducted using the PTB goniophotometer described in Sec. 2.3, which was calibrated against the PTB luminous intensity working standards immediately before the intercomparison. Each lamp was measured twice with the goniophotometer. One scan took approximately 30 min, and thus the burning time for each lamp was typically 1 h for measurements at PTB. The electrical instrumentation for lamp operation is similar to the one described for the luminous intensity measurements at PTB.

### 6.2 Results

Table 7 shows the results of the luminous flux measurements by the three laboratories. Each value is the result of two or more measurements with separate burnings of the lamp. Table 8 shows the ratios of the luminous flux values relative to the average of the three aboratories, together with the statistical uncertainty budget. The data are analyzed in the same manner as in Table 3, with the typical reproducibility (relative standard deviation of the mean) of measurements of the transfer lamps being 0.05 %. The relative expanded uncertainty, *U* (*k* = 2), of the results is estimated to be 0.22 %, higher than that of the luminous intensity re sults. Larger changes of all the transfer lamps, which were operated longer than the luminous intensity lamps, contributed to the increased uncertainty of comparison. The relative changes of lamps during the comparison (the last value minus the first value measured at the laboratory that prepared the lamp), as averaged in lamp groups, were −0.37 % (NIST lamps), +0.16 % (NPL lamps), and −0.22 % (PTB lamps), with a maximum difference of −0.49 %.

Table 9 shows the analysis of the lamp voltages measured by the three laboratories. All the laboratories used a four-pole socket to operate the transfer lamps and measure the lamp voltages. The data are analyzed in the same manner as in Table 4. All the lamps showed reasonable reproducibility during the intercomparison, and did not indicate any significant changes of their electrical characteristics. However, the estimated uncertainty of the comparison is larger than that in the luminous intensity comparison (Table 4). This explains the larger uncertainty of the luminous flux measurements. The differences in the voltage measurements among the three laboratories are not significant when compared with the uncertainty of the comparison and are largely due to the differences in measurement conditions as described earlier.

From the results, the relationships between the magnitudes of the luminous flux units at the three laboratories is: NIST lumen / NPL lumen = 1.0040, NPL lumen / PTB lumen = 0.9939, and PTB lumen / NIST lumen = 1.0022. With the stated relative expanded uncertainties (*k* = 2) of the luminous flux units of NIST, NPL, and PTB being 0.53 %, 0.35 %, and 0.6 %, respectively, the uncertainty bars of the three laboratories overlap with one another. The range of the variations of the magnitude of the units among the three laboratories (0.61 %) is a notable improvement over the results of the 1985 CCPR intercomparison (1.6 %) [1].

## 7. Conclusion

A trilateral intercomparison of photometric units maintained at NIST, NPL, and PTB was conducted. Seven luminous intensity transfer lamps, three transfer photometers, and eight luminous flux transfer lamps were circulated to compare the measured values of luminous intensity, luminous responsivity, and luminous flux. The measurements at NIST were based on the units realized in 1995. The measurements at NPL and PTB were based on their photometric units maintained since 1985 and compared through the CCPR in 1985.

The results showed that the relative luminous intensity values measured by NIST, NPL, and PTB were found to be 1.0014, 1.0021, and 0.9966, respectively, with respect to the average of the three laboratories, with a relative expanded uncertainty of 0.0014 (*k* = 2) for each value. The luminous responsivity of the photometers were measured by NIST, NPL, and PTB to be 0.9977, 0.9989, and 1.0035, respectively, with respect to the average of the three laboratories, with a relative expanded uncertainty of 0.0023 (*k* = 2) for each value. The reversed ratios (corresponding to illuminance) are 1.0023, 1.0011, and 0.9965, which is consistent with the results of the luminous intensity. The relative luminous flux values measured by NIST, NPL, and PTB were found to be 0.9994, 1.0034, and 0.9972, respectively, with respect to the average of the three laboratories, with a relative expanded uncertainty of 0.0023 (*k* = 2) for each value.

From the results, the relationship of the magnitude of the units between laboratories is; NIST candela/NPL candela = 1.0007, NPL candela / PTB candela = 0.9945, and PTB candela / NIST candela = 1.0048; NIST lumen / NPL lumen = 1.0040, NPL lumen / PTB lumen = 0.9939, and PTB lumen/NIST lumen = 1.0022.

The photometric units maintained at the three laboratories were found to agree within the stated expanded uncertainty of the realization of the units at the three laboratories. Most of the transfer lamps and photometers showed acceptable reproducibility, but some artifacts experienced significant changes, which will be studied for further improvements.

## Figures and Tables

**Table 1:** Scheme of the trilateral intercomparison

Date measured at	June 95 PTB	June 95 NIST	Nov 95 NPL	Nov 95 PTB	June 96 NPL	July 96 NIST	Sep 96 PTB	Oct 96 NIST	Mar 97 NPL	Nov 97 PTB
Intensity										
NIST lamps (3)		x	x	x	x	x	x	x		
NPL lamps (2)			x	x	x	x			x	
PTB lamps (2)	x		x	x	x	x			x	x
Responsivity										
NIST photometer		x	x	x	x	x				
NPL photometer			x	x	x	x			x	
PTB photometer	x		x	x	x	x			x	x
Flux										
NIST lamps (4)		x	x	x	x	x	x	x		
NPL lamps (2)			x	x	x	x			x	
PTB lamps (2)	x		x	x	x	x			x	x

**Table 2:** Results of the luminous intensity measurements

				Measured luminous intensity (cd)						
Lamp No.	NIST 6/95	NIST 6/96	NIST 10/96	NPL 11/95	NPL 6/96	NPL 3/97	PTB 6/95	PTB 11/95	PTB 9/96	PTB 11/97
NBS10011	966.85	967.87	967.12	966.61	966.99			963.19	963.89	
NBS10014	912.16	911.79	910.71	912.66	910.49			908.98	908.40	
NBS10015	948.68	950.38	949.87	950.71	949.45			947.02	946.90	
PA765		449.21		449.63	449.72	449.61		446.19		
PA789		443.44		443.82	444.03	444.24		440.38		
PTB644		234.14		233.90	233.99	234.40	232.30	232.64		233.07
PTB647		229.70		230.82	230.11	230.11	229.10	228.78		228.58

**Table 3:** Analysis of the luminous intensity comparison

Transfer lamp	Average of 3 Labs (cd)	NIST/ave.	*σ* of mean (%)	NPL/ave.	*σ* of mean (%)	PTB/ave.	*σ* of mean (%)	Average *σ*_1_ of mean (%)
NBS10011	965.87	1.0015	0.06	1.0010	0.06	0.9976	0.06	0.06
NBS10014	910.61	1.0010	0.07	1.0011	0.14	0.9979	0.06	0.09
NBS10015	948.89	1.0008	0.07	1.0012	0.07	0.9980	0.05	0.07
PA765	448.35	1.0019		1.0029	0.05	0.9952		0.05
PA789	442.62	1.0019		1.0032	0.06	0.9949		0.06
PTB644	233.64	1.0022		1.0020	0.08	0.9959	0.11	0.10
PTB647	229.62	1.0003		1.0032	0.11	0.9965	0.08	0.10
	Mean	1.0014		1.0021		0.9966		*σ*_2_
	*U* (*k* = 2)			0.0014				0.07

**Table 4:** Analysis of the lamp voltages in the luminous intensity comparison

Transfer lamp	Average of 3 labs (V)	NIST/ave.	*σ* of mean (%)	NPL/ave.	*σ* of mean (%)	PTB/ave.	*σ* of mean (%)	Average *σ*_1_ of mean (%)
NBS10011	83.73	1.0003	0.03	0.9999	0.00	0.9997	0.01	0.01
NBS10014	83.82	1.0002	0.04	1.0004	0.00	0.9994	0.03	0.02
NBS10015	83.50	1.0002	0.03	1.0001	0.01	0.9997	0.01	0.02
PA765	12.57	0.9976		0.9974	0.07	1.0051		0.07
PA789	12.58	0.9961		0.9988	0.22	1.0051		0.22
PTB644	28.83	1.0003		0.9998	0.01	0.9999	0.02	0.02
PTB647	28.87	1.0000		1.0001	0.01	0.9999	0.01	0.01
	Average	1.0002^a^		1.0001^a^		0.9998^a^		*σ*_2_
	*U* (*k* = 2)			0.0003				0.02^a^

a NPL lamps excluded from averaging the results.

**Table 5:** Results of the luminous responsivity measurements

Photometer No.	6/95		Luminous responsivity (nA/lx)								
		NIST	Average	11/95	NPL		Average	6/95	PTB		Average
		7/96			6/96	3/97			11/95	11/97	
NIST No. 2	2.3158	2.3115	2.3136	2.3157	2.3177		2.3167		2.3258		2.3258
NPL 58218		14.644	14.644	14.705	14.687	14.613	14.668		14.729		14.729
PTB LV10B		3.3811	3.3811	a	3.3852	3.3820	3.3836	3.3990	3.4030	3.4030	3.4017

a The photometer was set-up incorrectly at NPL and no data are given for this entry.

**Table 6:** Analysis of the luminous responsivity comparison

Transfer photometer No.	Average of 3 labs (nA/lx)	NIST/ave.	*σ* of mean (%)	NPL/ave.	*σ* of mean (%)	PTB/ave.	*σ* of mean (%)	Average *σ*_1_ of mean (%)
NIST No. 2	2.3187	0.9978	0.11	0.9991	0.07	1.0031		0.09
NPL 58218	14.680	0.9975		0.9992	0.20	1.0033		0.20
PTB LV10B	3.3888	0.9977		0.9985	0.06	1.0042	0.06	0.06
	Mean	0.9977		0.9989		1.0035		*σ*_2_
	*U* (*k* = 2)			0.0023				0.12

**Table 7:** Results of the luminous flux measurements

Lamp No.	6/95		8/97	11/95	Measured luminous flux (lm)					
		NIST			NPL			PTB		
		7/96			6/96	3/97	6/95	11/95	9/96	11/97
NBS8382	2228.3	2219.8		2237.0	2235.3			2218.4		
NBS8385	2281.5	2270.3		2290.0	2285.2			2267.4		
TF4-11	475.9	475.1	474.9	477.1	476.3			474.0	473.5	
TF4-12	467.9	466.1	466.1	469.0	466.7			465.5	464.9	
NPL-206		3313.6		3330.0	3333.9	3334.0		3312.3		
NPL-298		3916.5		3932.0	3936.9	3939.7		3911.4		
PTB-3		894.2		899.5	895.6	896.9	894.3	893.9		891.8
PTB-19		1149.4		1152.9	1148.5	1151.4	1148.0	1147.3		1146.2

**Table 8:** Analysis of the luminous flux comparison

Transfer lamp	Average of 3 labs (lm)	NIST/ave.	*σ* of mean (%)	NPL/ave.	*σ* of mean (%)	PTB/ave.	*σ* of mean (%)	Average *σ*_1_ of mean (%)
NBS8382	2226.2	0.9990	0.20	1.0045	0.06	0.9965		0.13
NBS8385	2277.0	0.9995	0.25	1.0047	0.12	0.9958		0.18
TF4-11	475.3	1.0001	0.08	1.0030	0.10	0.9968	0.07	0.09
TF4-12	466.6	1.0002	0.14	1.0027	0.25	0.9970	0.08	0.16
NPL-206	3319.5	0.9982		1.0040	0.06	0.9978		0.06
NPL-298	3921.4	0.9988		1.0038	0.08	0.9975		0.08
PTB-3	894.9	0.9991		1.0027	0.14	0.9982	0.10	0.12
PTB-19	1149.2	1.0002		1.0016	0.12	0.9983	0.07	0.10
	Mean	0.9994		1.0034		0.9972		*σ*_2_
	*U* (*k* = 2)			0.0022				0.11

**Table 9:** Analysis of the lamp voltages in the luminous flux comparison

Transfer lamp	Average of 3 labs (V)	NIST/ave.	*σ* of mean (%)	NPL/ave.	*σ* of mean (%)	PTB/ave.	*σ* of mean (%)	Average *σ*_1_ of mean (%)
NBS8382	92.827	1.0003	0.02	0.9994	0.09	1.0003		0.06
NBS8385	93.696	1.0004	0.00	0.9995	0.07	1.0001		0.04
TF4-11	23.814	1.0003	0.03	0.9994	0.08	1.0003	0.01	0.04
TF4-12	23.908	1.0003	0.04	0.9994	0.06	1.0003	0.01	0.04
NPL-206	100.14	1.0003		0.9996	0.06	1.0001		0.06
NPL-298	120.79	1.0002		0.9995	0.08	1.0003		0.08
PTB-3	19.717	1.0002		0.9997	0.04	1.0001	0.01	0.02
PTB-19	21.082	1.0004		0.9996	0.04	1.0000	0.01	0.03
	Mean	1.0003		0.9995		1.0002		*σ*_2_
	*U* (*k* = 2)			0.0009				0.04

## Footnotes

1 As an aid to the reader, the appropriate SI unit in which a quantity should be expressed is indicated in parenthesis when the quantity is first introduced.

2 Certain commercial equipment, instruments, or materials are identified in this paper to foster understanding. Such identification does not imply recommendation or endorsement by the National Institute of Standards and Technology, nor does it imply that the materials or equipment identified are necessarily the best available for the purpose.
